# Analysis of Prehospital Care of Migrants Who Arrive Intermittently at the Coasts of Southern Spain

**DOI:** 10.3390/ijerph17061964

**Published:** 2020-03-17

**Authors:** José Antonio Ponce-Blandón, Tatiana Mérida-Martín, Maria del Mar Jiménez-Lasserrotte, Nerea Jiménez-Picón, Juana Macías-Seda, Maria de las Mercedes Lomas-Campos

**Affiliations:** 1 Centro Universitario de Enfermería de la Cruz Roja, Universidad de Sevilla, 41009 Sevilla, Spain; nejipi@cruzroja.es; 2 Servicios de Asistencia Médica de Urgencias (SAMU), 29006 Málaga, Spain; ta.merida@gmail.com; 3 Departamento de Salud y Socorros. Asamblea Provincial de Cruz Roja de Almería, 04002 Almería, Spain; marjil@cruzroja.es; 4 Facultad de Enfermería, Fisioterapia y Podología. Universidad de Sevilla, 41009 Sevilla, Spain; jmseda@us.es (J.M.-S.); mlomas@us.es (M.d.l.M.L.-C.)

**Keywords:** emigration and immigration, coasts (coastline), pathology, preospital care

## Abstract

*Background*: The aim of this study is to identify the sociodemographic characteristics and the most frequent diseases and nursing interventions carried out on migrants arriving by sea at southern Spain. *Method*: Cross-sectional, descriptive, and retrospective study based on the database of the Spanish Red Cross Intervention Activation System. All migrants who arrived on the coasts of a southern province during 2016 and were assisted by the Red Cross were included. *Results*: A total of 2027 people were registered, mostly males, aged between 18 and 40 years. Of these, 4.9% required healthcare, and 2.9% were referred to hospital. Highlighted diagnoses were headaches (15.6%), pregnancy (12.8%), and lower-limb wounds (6.4%), and outstanding nursing interventions were “care of wounds” (24.7%), “pain management” (21.9%), and “prenatal care” (15.2%). Statistically significant relationships were found between the diagnosed diseases and gender, geographic area of origin, and seasonal time of the year, as well as between nursing interventions performed and those three variables. *Conclusions*: Although in general, a good health condition was observed in most of the migrants treated, the most frequent health situations attended were related to dermatological, gynecological, and headache problems. The most performed nursing interventions were related to skin/wound care and promotion of physical comfort, requiring a low need for hospital transfers. Female gender, origin from sub-Saharan Africa and arrival in the summer period carry a greater risk of suffering health problems when migrants reach Spanish coasts.

## 1. Introduction

The movements of migrants have increased significantly in the last 20 years in Spain, with the aim of settling in Europe and having better living conditions than in the country of origin [1,2,3], making the Andalusian coast one of the main entry points, thanks to the protection system offered by our immigration policies, among other reasons [1]. During the last few years, the migratory routes followed by the African population to reach European land have changed due to the border blockade, difficulties embarking from the country of origin, or the dangerousness of the maritime routes, with them devising alternatives to circumvent European border control [4]. As of 2006, the Canary Islands began to be the coast with the greatest influx of maritime immigration [2] as a consequence of a greater border control in the natural route of the Strait of Gibraltar, but the Integrated External Surveillance System (SIVE) was also located in Ceuta, Melilla and the Canary Islands, with the Strait of Gibraltar becoming a location for the displacement of migrants once again [4], although due to the intensification of surveillance, this time the routes have been diverted to the coast of Granada and Almería [1,4]. 

The right to healthcare of those who arrive on the Spanish coast must be guaranteed and those from the most vulnerable groups [5], that is, those needing emergency care, pregnant women, and minors, must receive particular attention and there are regulations and specific action plans for these situations [6,7,8]. However, prehospital care is usually covered by non-governmental organizations such as the Red Cross, which offers a variety of assistance to migrants and refugees in coordination with the public health system [9].

The greater scientific production at national and international level focuses on later phases of the migration process, so that there are few descriptive studies about the healthcare received by migrants upon their arrival, with the noteworthy studies being those that describe the relationship of different sociodemographic factors and psychological factors that cause mental disorders, level of discrimination and stress [10,11,12], or social and labor integration problems [13].

The specific works on prehospital emergency care provided to the migrant population that enter our country by sea are limited to the Canarian context [14,15,16]. Therefore, in the study of Matos-Castro and Padrón-Peña [15] more than 17,000 migrants were studied over a year, generally presenting a good health condition and identifying difficulties when it came to anamnesis and diagnosis caused by the language and cultural barrier. The work of Rodríguez del Rosario et al. [16], with a larger sample of 19,000 migrants, found a low proportion of hospital referrals, most of them by specific diagnostic procedures to detect infectious or parasitic diseases, although serious situations such as deaths caused by the electrolyte imbalance secondary to the long journey were also detected. The editorial by Hernández-Sánchez et al. [14] emphasizes the reception, assistance, and classification at the beach or port, as well as the need for coordination between non-governmental organizations (NGOs) such as the Red Cross and the public health system, paying special attention to the existence of sunken boats and the arrival of unaccompanied minors. 

Despite the information available on the morbidity and mortality of the migrant population, it is necessary to continue carrying out research that offers a view of the crisis of irregular maritime migration from the point of view of accident and emergency. The southern Spanish coasts are an essential scenario in understanding the in situ care and the possible health complications of those arriving from North Africa. It is necessary to increase the evidence centered on the most probable diseases after the migratory process by sea and the risk of secondary complications, which could have repercussions in the form of a better functioning and a quick response to emergency situations. In the same way, knowing the coordinated health interventions among the medical and nursing professionals could be relevant to complement the direct assistance with the promotion, prevention, and rehabilitation of the health of immigrants from a transcultural perspective [17].

For all this, a study is proposed to identify the most frequent diseases diagnosed in the migrant population that arrives at the coasts of the south of Spain and to inquire about the prehospital healthcare actions and the nursing interventions carried out at the coast, as well as the hospital referrals required, also exploring the possible relationships between sociodemographic variables and care variables.

## 2. Materials and Methods

A descriptive, observational, and retrospective design study was carried out. It included, without any exclusion, the entire information about the migrant population arriving on boats at the coasts of the province of Almería (a province located in the southeast of the country with 219 kilometers of Mediterranean coastline) that had been cared for by the Spanish Red Cross teams during the period of time from 1 January, 2016 until 31 December, 2016. Sociodemographic variables (age, gender, country of origin) and care variables were included as study variables: date of arrival, hospital referrals, medical diagnoses included in the international statistical classification of diseases (CIE-10) [18], and nursing interventions based on the Nursing Interventions Classification (NIC) [19]. Given their special incidence, the episodes of headache were treated as a specific category in the groups of diseases.

The source of information used was the Spanish Red Cross Intervention Activation System of the Provincial Assembly of the Red Cross of Almería, which includes the actions and the assistance variables that describe the interventions. The data collection process was developed by extracting the digitized data related to the study variables through the Department of Health and Relief of the aforementioned Provincial Assembly and by collecting the health interventions carried out by the intervention teams that were manually annotated by them in the Emergency Intervention Sheets in situ. The database was refined by cancelling those fields that were not complete or lacking information relevant to the objectives of the study, and all records collected were kept.

For the analysis of the data, some variables were recoded by grouping those categories that facilitated the analysis without losing relevant information. For the qualitative variables, relative and absolute frequencies were calculated, with 95% confidence intervals and, for continuous variables, measures of central tendency and dispersion were calculated. We analyzed the bivariate relationships between sociodemographic variables and care variables in an exploratory manner using the chi-square test, and a statistically significant relationship when the value of *p* was below 0.05 was recognized. Due to the small number of expected frequencies in some of the variable categories, the Yates correction was applied to improve the consistency of the test. The analysis of the data was carried out with the EpiInfo™ 7.2 statistical program (Center for disease control and prevention, Atlanta, GA, USA).

Regarding the ethical aspects of the study, authorization to carry out the study was obtained from the Autonomous and Provincial Coordinator of the Spanish Red Cross. Researchers had restricted access to personal information of any kind by means of an encryption of personal data. This involved the assignment of numerical codes to each participant from the Red Cross organization, enabling the researchers exclusively the access to the information of variables of study, hiding their most sensitive identification data, such as the name of the migrant or the identification number assigned by the police upon arrival.

Throughout the data collection process, the ethical principles for medical research in humans described in the latest revision of the Declaration of Helsinki carried out in Brazil 2013 [20] were applied. Furthermore, access and analysis of the data from participants included in this study was expressly authorized by the Provincial Office of Red Cross in Almería, in accordance with the authorization dated 4 July, 2017. It also was aproved by Research Ethics Committee from Spanish Red Cross Nursing University College, in accordance with document 05/2017 dated 15 september, 2017.

## 3. Results

The study population consisted of information from all the migrant population, in total 2207 migrants cared for by the Red Cross, of which 91.2% were males (*n* = 2013). The people cared for came from a total of 149 different boats, comprising an average of 14.8 passengers (SD 11.9), with boats carrying between 6 and 15 passengers being the most frequent (30.2%, *n* = 45). The season of the year in which a greater influx was observed was the summer, in which 34.2% of the total number of boats arrived (*n* = 756). 

The age range of the population cared for was between 0 and 53 years, with a mean of 22 years (SD = 6.3). The majority of the population was between 18 and 40 years old, accounting for 70.5% of the total population (*n* = 1557). 

By country of origin, the most frequently registered country was Algeria, which represented 40.4% of the total (*n* = 893), followed by Ivory Coast, with 16.9% (*n* = 374). By geographical areas, immigrants from sub-Saharan Africa were the most frequent, since 53.1% of the total (*n* = 1172) came from these countries. The detail of all these sociodemographic variables can be seen in Table 1.

Regarding the care variables, it was observed that 95.1% (*n* = 2908) did not present any health problem, compared to 4.9% (*n* = 109) that was diagnosed with a health problem. Of the great variety of diseases that were diagnosed by health teams, the most frequent health states were headaches with 15.6% (*n* = 17), pregnancy with 12.8% (*n* = 14), and lower-limb wounds with 6.4% (*n* = 7). There were a large number of diseases that were only identified in one or two people. Once the diseases were grouped according to specialties, the most frequent health problems were dermatological problems with 24.7% of the total (*n* = 27), followed by gynecological problems with 21.1% (*n* = 23), and headache episodes, with 15.6% (*n* = 17). Sixty-five patients were transferred to three different hospitals, making up 2.9% of the population cared for (*n* = 65). Torrecárdenas Hospital received the vast majority of transfers, with 95.3% of referrals (*n* = 62). 

Nursing interventions were registered in a total of 105 people cared for. The most carried out were “care of wounds”, in 24.7% of the total of people cared for (*n* = 26); “pain management”, in 21.9% (*n* = 23); and “prenatal care” with 15.2% (*n* = 16). Once grouped in their respective classes, the most performed intervention was “skin/wound care”, with 24.7% of the total people cared for (*n* = 26), followed by the “promotion of physical comfort”, which represented 23.8% (*n* = 25). The distribution of medical diagnoses with their groupings, as well as the casuistry of hospital transfers and nursing interventions with their grouping by classes, can be seen in Table 2.

Statistically significant differences were found by gender, since 33.5% (*n* = 65) of women were diagnosed with any disease, versus 2.58% (*n* = 52) of men (*p* < 0.0001). Dermatological diseases were the most diagnosed diseases in men (30.7%, *n* = 16) versus gynecological causes (35.3%; *n* = 23) in women (*p* = 0.0001). Nursing interventions were also performed more frequently in women (29.9%, *n* = 55 in women vs. 2.4%, *n* = 49 in males) (*p* < 0.0001), with most outstanding intervention among women being “care of a new baby” (29.1%, *n* = 16), compared to the most frequent intervention in males, which was “skin/wound care” (36.7%; *n* = 18) (*p* = 0.0002).

Regarding the age groups, no differences were found between immigrants over 18 years old and those under 18, neither in terms of the frequency of diagnoses (*p* = 0.886) nor in terms of nursing interventions (*p* = 0.880). However, significant differences were found regarding the types of diseases between both age groups, highlighting dermatological diseases in minors (32.3%, *n* = 10) versus gynecological diagnoses in adults (26.5%; *n* = 22) (*p* = 0.0123). In the same way, regarding nursing interventions, the number of “skin/wound care” interventions in minors (31.0%; *n* = 9) was the highest in that group, versus “promotion of comfort” as the most frequent intervention in adults (26.0%, *n* = 19) (*p* = 0.024). 

Regarding the differences according to the geographical area of origin of the migrant, the sub-Saharan population had a higher overall frequency of diseases (7.3%, *n* = 86) versus migrants coming from Maghreb (3.0%, *n* = 31) (*p* < 0.0001). Likewise, the sub-Saharan population was subject to more nursing interventions (6.5%; *n* = 76) versus migrants from Maghreb (2.7%, *n* = 28) (*p* < 0.0001). However, no significant differences were found in relation to type of diagnoses or nursing interventions.

Furthermore, statistically significant differences were found in the seasonal distribution of diagnoses since a greater number of diseases was observed in summer (6.8%, *n* = 52) compared to the rest of the seasons (*p* = 0.0262). Seasonal differences were also found regarding nursing interventions, which again were more frequent in summer (6%, *n* = 46) (*p* = 0.0080). However, no differences were found regarding the types of diagnoses performed in each season, although in relation to the type of nursing interventions, differences were found between the seasons (*p* = 0.0452).

Table 3 shows the detail of the results of the bivariate analysis between sociodemographic and care variables.

## 4. Discussion

Some limitations are identified that make it advisable to be prudent when interpreting the results due to the characteristics of the database of the care activity of the Red Cross, since this has not been specifically designed for the research, which involved an attempt to adapt the information to the objectives of the work. Likewise, the registry of care variables was completed by different users at different times, which has demonstrated some inconsistencies although attempts have been made to alleviate this by a thorough prior review of the records. Although categories of variables were regrouped with an excessive number of categories and this could have favored some classification bias, the groupings were carried out following standardized criteria [21], based on similar work [15,16]. The identified diagnoses were grouped according to the specialty to which they belong [18], excluding headache that was established as an independent category due to its high frequency. In relation to representativeness and sample size, its high quality and large size stand out thanks to the fact that the database included 100% of the population assisted by the Red Cross care teams throughout the year, so that sampling was not necessary, therefore avoiding selection biases and reinforcing the validity of the study. 

In relation to the results, it should be considered that the reasons why the African population migrates to European countries include achieving a better quality of life and job search [22], behaviors that are attributed to the male role in their countries of origin and culture, although, in the case of Spain, the fact that it is the gateway to southern Europe to access other countries is also added [1,2,3]. This explains why the bulk of the population is male and between 18 and 40 years old, a profile that is more likely to enter the labor market in their cultures. However, it is noteworthy that 7.2% of women were pregnant, which would explain the search for the nationality of their future children, but for the rest of women, the reasons can be multiple and varied: from meeting with husbands and families that have emigrated before, to the search for jobs [23]. 

Anyway, although the migrant women identified in this study are quantitatively considerably less than men attended, women irregular migrants who arrive in Spain by small boat have specific needs and healthcare problems, as the statistically found differences by gender in this study demonstrated, in line with other studies that highlight the need for detecting weaknesses and promoting screening and safety protocols focused on women irregular migrants, considering, in addition, that sadly many irregular migrants women are an object of sexual exploitation and that the mother–child dyad turns out to be the axis in human tracking [24].

However, the presence of minors is another aspect to consider, including the so-called “Accompanied Child Irregular Migrants” (babies and older children), given the paradigm of vulnerability related to the fact that crossing the sea is for them “playing with death” and that priority specific care for them is required, identifying high-risk situations [25].

The good weather conditions of the Mediterranean during the summer meant that more boats were detected in this season, along with a greater demand for employment in these months, becoming a stimulus for a greater influx. However, summer was also significantly the season of the year in which more medical diagnoses and more interventions of nursing were performed. Perhaps the possible “pull effect” of the good weather is a double-edged sword because high summer temperatures can lead to serious problems of dehydration and heat stroke, as other studies in other high-temperature contexts demonstrate [26,27], although no significant differences were specifically found between the diagnosed diseases or nursing interventions, in this case.

The number of people who embarked on the same boat should be noted, taking into account the poor quality of these boats and their small size, meaning that the trips are carried out in unhealthy conditions and conditions of overcrowding. The insecurity in the boats is an aspect of great importance, because the sunken boats are a real risk that the care teams must assume [14,15,28]. In fact, there are hundreds of migrant men, women, and children, who die every year in their attempt to cross the Mediterranean Sea to reach Europe, sometimes even being unidentified, leaving this fundamental right unfulfilled [29]. 

Another aspect that has drawn attention to the results of our study are the differences found in the presence of health problems and in the interventions carried out depending on the region of the countries of origin of immigrants. Indeed, immigrants from sub-Saharan African countries had more than twice as many health problems as immigrants from North Africa. This can be explained because sub-Saharan immigrants travel much longer (sometimes thousands of miles for several months) before reaching the coasts of North Africa to cross the Strait of Gibraltar [22], which leads to more intense exposure and prolonged health risks, although there are also studies that prove that in countries of sub-Saharan Africa there is a higher incidence of infectious diseases [30], so if they carry these diseases from their departure, this could also explain a part of these differences.

But in any case, clearly the majority of the population cared for did not have health problems. Of the diseases diagnosed, headaches and wounds were highlighted, which could have occurred both on the boat and before journeys were started. Headache can be related to the circumstances of the trips, which can entail dehydration, the crowding of people inside the boat, and prolonged exposure to the sun [15,22,31]. For the most part, non-traumatic wounds corresponded to superficial wounds, which may also be related to the nature of the journey [15]. This fact also contributes to the fact that, when regrouping the diagnoses by systems, the most frequent diseases were dermatological ones, since both wounds and burns (solar or chemical by exposure to tetraethyl lead, a caustic compound that is formed when fuel from engines and seawater mix) [32] would correspond to the same category. In relation to hospital referrals, the low percentage of referral stands out, which is also related to the low frequency of severe diseases detected. 

Divergences and similarities are observed when comparing the results of this study with similar ones. For instance, in a cross-sectional study performed in the center for migrants of Lampedusa (Italy), attending migrants crossing the so-called “central Mediterranean route” [33] the most frequent diagnosis was scabies, skin infections, pediculosis and dermatitis, and also respiratory infections and varicella were the most represented infectious diseases, just quite different from the problems identified in this study, perhaps due to the specific characteristics of this route, very different from the route of the Strait of Gibraltar. Moreover, as mentioned, health problems related to exposure to high temperatures are especially frequent in illegal migratory movements such as across the Mexico–Arizona border [26,27] or, for example, it has been reported in immigrants who illegally access other countries in northern Europe such as the Netherlands, seeking care most frequently for injuries and dental problems [34]. Other studies also highlighted the mental health problems that have been identified among humanitarian migrants who access countries such as Australia where premigration potentially traumatic events and postmigration stressors were positively associated with posttraumatic stress disorder and severe mental illness [35], aspects that we have not detected in our study. In any case, the differences in the paths made by migrants analyzed in the different studies and, of course, the sociodemographic differential characteristics of the migrants, depending specially on their ethnicity and/or their country of origin, clearly mark the differences between the results found and the results of these other studies.

Comparing with studies developed in similar contexts, the study conducted in the Canarian archipelago [16] showed a distribution of health problems different from that observed in this work, since febrile syndromes or gastrointestinal disorders were more frequent, and some cases of malaria and electrolyte imbalances that required urgent hospitalization were identified. In addition, only 4.9% of the total registered population had health problems on the coasts of Almería, while in the Canary Islands 38.5% of the total required care. However, in another study conducted in Tenerife [15], only 3.7% of the total required healthcare, a percentage that is more similar to that observed in Almería. Hypothermia and hypoglycemia were identified as more frequent problems, as well as minor injuries and erosions, and hospital referral of all women and children was carried out to perform a gynecological and pediatric evaluation. 

There are more studies that describe the hospital care of immigrants residing in Spain [36,37,38] in a more extensive way, but these do not address the healthcare needs when the boats arrive. In the same way, other studies address these same issues related to hospital care or access to specific services offered by different health systems in the context of other countries, analyzing their barriers and difficulties [39,40,41,42]. These studies emphasize that the attention needed by these populations are not limited to those presented at the time of their arrival, but, sometimes, the approach to the problems that appear later are much more complex. All these studies are an absolute necessity, since crisis management establishes relief as a fundamental pillar, along with organization and logistics [43], and a greater number of studies of this type will provide the capacity to determine the assistance needs that are required. 

## 5. Conclusions

In conclusion, it has been possible to observe the healthcare circuit carried out, covering the interventions and diagnoses made at the coast and the required hospital referrals. During 2016, in general, a good health condition was observed in most of the migrants attended when they arrive to the coasts of Almería, requiring a low need for hospital transfers. Nevertheless, the most frequent health situations attended were related to dermatological, gynecological, and headaches problems, and in tune with this, the most performed nursing interventions were related to these diseases and health situations, such skin/wound care and promotion of physical comfort. A significant relationship has been proved between the diagnosed diseases and gender, geographic area of origin and seasonal time of the year, as well as between nursing interventions performed and those three variables. Thus, female gender, origin from sub-Saharan Africa, and arrival in summer period carry a greater risk of suffering health problems when migrants reach Spanish coasts.

The knowledge of these sociodemographic and care characteristics will enable health professionals to improve care in future situations of humanitarian action through a greater adaptation of action protocols and monitoring of these populations. It is necessary to carry out more studies of this nature given the high frequency of immigrants that arrive annually on our coasts, highlighting the important role that the Red Cross organization can play in it, with access to 100% of the irregular migrant population that arrive in our country through these means. The a systematized protocol would help to develop higher-quality care and guarantee more efficient humanitarian aid and especially useful are the findings revealed by this study in order to improve health planning and to ensure a more effective response to the health needs presented by immigrants crossing the Mediterranean Sea upon arrival at the main gate of Europe after this risky and sad journey.

## Figures and Tables

**Table 1:** Descriptive sociodemographic variables of the immigrant population cared for on the coasts of Almería.

**Gender**	**Absolute Frequency (*n*)**	**Percentage (95% CI)**	
Male	2013	91.2 (89.9–92.3)	
Woman	194	8.8 (7.6–10.0)	
Total	2207	100	
**No. of Passengers Per Vessel**	**Absolute Frequency (*n*)**	**Percentage**	**Accumulated Percentage**
1 passenger	13	8.7%	8.7%
2–5 passengers	32	21.4%	30.1%
6–15 passengers	45	30.2%	60.3%
16–25 passengers	28	18.8%	79.1%
26–40 passengers	26	17.4%	96.6%
More than 40 passengers	5	3.3%	100%
Total vessels	149	Range = (1–55)Average per vessel = 4.8 (SD = 11.9)	
**Seasonal Distribution**	**Absolute Frequency (*n*)**	**Percentage (95% CI)**	
Summer	756	34.2 (32.3–36.2)	
Autumn	680	30.8 (28.9–32.7)	
Spring	412	18.6 (17.1–20.3)	
Winter	359	16.2 (14.8–17.8)	
Total	2207	100	
**Age Range**	**Absolute Frequency (*n*)**	**Percentage (95% CI)**	**Accumulated Percentage**
0–24 months	8	0.3 (0.2–0.7)	0.3
2–13 years	24	1.1 (0.7–1.6)	1.4
14–17 years	573	25.9 (24.1–27.8)	27.3
18–40 years	1557	70.5 (68.6–72.4)	97.8
41–64 years	16	0.7 (0.4–1.1)	98.6
Undefined	29	1.3 (0.9–1.8)	100
Total	2207	Range = (0–53)Average age = 22 (SD = 6.3) Mode = 17	
**Country of Origin**	**Absolute Frequency**	**Percentage (95% CI)**	
Algeria	893	40.4 (38.4–42.5)	
Ivory Coast	374	16.9 (15.4–18.5)	
Guinea	198	8.9 (7.8–10.2)	
Cameroon	160	7.2 (6.2–8.4)	
Gambia	138	6.2 (5.3–7.3)	
Morocco	109	4.9 (4.1–5.9)	
DR Congo	75	3.3 (2.3–4.1)	
Burkina Faso	56	2.5 (1.9–3.2)	
Guinea-Bissau	41	1.8 (1.3–2.5)	
Mali	21	0.9 (0.6–1.4)	
Mauritania	17	0.7 (0.4–1.2)	
Sierra Leone	13	0.5 (0.3–1.0)	
Kenya	12	0.5 (0.3–0.9)	
Eritrea	11	0.5 (0.2–0.9)	
Central African Republic	9	0.4 (0.2–0.7)	
Syria	9	0.4 (0.2–0.7)	
Togo	8	0.3 (0.1–0.7)	
Ghana	7	0.3 (0.1–0.6)	
Liberia	7	0.3 (0.1–0.6)	
Uganda	6	0.2 (0.1–0.6)	
Comoro Islands	5	0.2 (0.1–0.5)	
South Sudan	5	0.2 (0.1–0.5)	
Other countries ^a^	33	14.9 (11.1–18.3)	
Total	2207	100	
**Geographic Area of Origin**	**Absolute Frequency (*n*)**	**Percentage (95% CI)**	
Sub-Saharan Africa	1172	53.1 (51.0–55.2)	
Maghreb countries	1021	46.2 (44.2–48.3)	
Asian countries	13	0.6 (0.3–1.0)	
Other	1	0.05 (0.01–0.2)	
Total	2207	100	

^a^ Countries of origin from which less than five immigrants were cared for: Chad, Sudan, Palestine, Angola, Benin, Niger, Armenia, Cape Verde, Egypt, Ethiopia, Gabon, Libya, Nigeria, Senegal, Somalia, Tanzania, Zambia, and Zimbabwe.

**Table 2:** Descriptive outcomes of care variables (diagnoses, hospital referrals, and nursing interventions) in the migrant population cared for on the coasts of Almería.

**Diagnoses**	**Absolute Frequency (*n*)**	**Percentage (95% CI)**
Non-specific headache and/or facial pain	17	15.6 (9.3–27.9)
Pregnancy (state of)	14	12.8 (7.2–20.6)
Unspecified and/or multiple wound/s of the lower limb	7	6.4 (2.6–12.7)
Candidiasis of unspecified localization	4	3.6 (1.0–9.1)
Sciatica or lumbosciatica	4	3.6 (1.0–9.1)
Dyspepsia and/or gastralgia	4	3.6 (1.0–9.1)
Unspecified upper respiratory tract diseases	3	2.7 (0.5–7.8)
Fever	3	2.7 (0.5–7.8)
Unspecified and/or multiple wound/s of the upper limb	3	2.7 (0.5–7.8)
Degree burn of the leg and unspecified site	3	2.7 (0.5–7.8)
Solar dermatitis (sunburn, solar erythema)	2	1.8 (0.02–6.4)
Dermatitis and eczemas of unspecified cause	2	1.8 (0.02–6.4)
Non-specific abdominal pain (excludes renal colic)	2	1.8 (0.02–6.4)
Ankle sprain	2	1.8 (0.02–6.4)
Unspecified fracture/s of the upper limb, closed	2	1.8 (0.02–6.4)
Abdominal hernia	2	1.8 (0.02–6.4)
Malaise and/or fatigue	2	1.8 (0.02–6.4)
Multiple second-degree burns (blisters, epidermal loss)	2	1.8 (0.02–6.4)
Common cold	2	1.8 (0.02–6.4)
Other diagnoses ^a^	29	26.6 (17.8–35.6)
Total	109	100
**Grouping of Diagnoses**	**Absolute Frequency (*n*)**	**Percentage (95% CI)**
Dermatological	27	24.7 (15.7–31.7)
Gynecological	23	21.1 (12.8–28.0)
Headaches	17	15.6 (8.7–22.2)
Other	11	10.1 (4.5–15.1)
Traumatology	9	8.2 (3.6–14.1)
Respiratory	7	6.4 (2.5–12.0)
Gastrointestinal	6	5.5 (1.9–10.8)
Non-traumatic osteomuscular	6	5.5 (1.9–10.8)
Optical	3	2.5 (0.5–7.3)
Total	109	100
**Transfers to Hospitals**	**Absolute Frequency (*n*)**	**Percentage (95% CI)**
Hospital Torrecárdenas	62	95.3 (87.1–99.0)
Hospital Provincial	2	3.0 (0.3–10.6)
Hospital Toyo	1	1.5 (0.04–8.3)
Total	65	100
**Nursing Interventions (NIC)**	**Absolute Frequency (*n*)**	**Percentage (95% CI)**
Care of wounds	26	24.7 (16.8–34.1)
Pain management	23	21.9 (14.4–31.0)
Prenatal care	16	15.2 (8.9–23.5)
Identification of risks	11	10.4 (6.2–17.3)
Treatment of fever	8	7.6 (3.9–13.2)
Infection control	5	4.7 (1.5–10.7)
Immobilization	4	3.8 (1.0–9.5)
Other ^b^	12	11.4 (6.6–18.1)
Total	105	100
**NIC Grouping**	**Absolute Frequency (*n*)**	**Percentage (95% CI)**
Skin/wound care	26	24.7(16.8–34.1)
Promotion of physical comfort	25	23.8 (16.4–33.1)
Risk management	17	16.1 (9.7–24.6)
Care for a new baby	16	15.2 (8.9–23.5)
Thermoregulation	10	9.5 (4.6–16.8)
Immobility control	4	3.8 (1.0–9.4)
Control of electrolytes and acid-base	2	1.9 (0.23–6.7)
Other ^c^	5	4.7 (1.8–10.6)
Total	105	100

^a^ Other diagnoses that were recorded in a single occasion: Unspecified abortion, anxiety, muscle contracture, breast contusion, chest wall contusion, lower-limb contusion with unspecified location, pain in or around the eye, generalized pain, chills, hypothermia not associated with low ambient temperature, unspecified chest pain, rash on legs, knee sprain, pharyngitis, foot fracture (tarsus and metatarsus), closed, heat stroke/sun stroke, injury to the genital organs, including traumatic amputation, wound of abdominal wall and/or breast and/or trunk, inguinal hernia, non-specific hyperglycemia, non-specific hypoglycemia, acute upper respiratory infection of multiple or unspecified localization (excludes influenza), unspecified local infection of the skin and subcutaneous tissues, nausea with vomiting (excludes hematemesis), bloodshot eye or suppuration of the eye, unspecified corneal opacity, pruritus, degree burn to the upper limb (except wrist and hand) and unspecified site, burn to the foot of unspecified degree, degree burns to the trunk and unspecified site, vomiting only. ^b^ Other nursing interventions that were recorded on a single occasion: administration of topical medication, control of bleeding, reduction of anxiety, breast examination, management of diarrhea, management of hyperglycemia, management of hypoglycemia, management of nausea, management of pruritus, maintenance in family processes, treatment of exposure to heat, and treatment of hypothermia. ^c^ Other groups of Nursing Interventions Classification (NIC) interventions applied in a single occasion: drug control, control of removal, control of tissue perfusion, care of life, and promotion of psychological comfort.

**Table 3:** Bivariate analysis results between sociodemographic variables and care variables. Migrant population cared for on the coasts of Almería.

Bivariate Analysis	Frequency by Diagnoses or Nursing Interventions Performed % (*n*)	Risk Ratio (95% CI)	Statistical Value, Degree of Freedom, Level of Significance	Frequency by Type of Diagnoses or Nursing Interventions. Most Frequent Category % (*n*)	Statistical Value, Degree of Freedom, Level of Significance
Gender vs. Diagnoses of any disease	Males 2.58% (52)	12.9 (9.3–18.1)	χ² = 337.01 df = 1 *p* < 0.0001 (*)	Dermatological 30.7% (16)	χ² = 24.92 df = 4 *p* = 0.0001 (*)
	Women 33.5% (65)			Gynecological 35.3% (23)	
Gender vs. Nursing Interventions	Males 2.4% (49)	12.2 (8.6–17.4)	χ² = 281.36 df = 1 p < 0.0001 (*)	Skin/wound care 36.7% (18)	χ² = 21.88 df = 4 *p* = 0.0002 (*)
	Women 29.9% (55)			Care for a new baby 29.1% (16)	
Age group vs. Diagnoses	<18 years 5.1% (31)	0.97 (0.6–1.4)	χ² = 0.0205 df = 1 *p* = 0.886 (ns)	Dermatological 32.3% (10)	χ² = 12.80 df = 4 *p* = 0.0123 (*)
	≥18 years 5.3% (83)			Gynecological 25.5% (22)	
Age group vs. Nursing Interventions	<18 years 4.8% (29)	1.03 (0.6–1.5)	χ² = 0.0022 df = 1 *p* = 0.880 (ns)	Skin/wound care 31.0% (9)	χ² = 11.23 df = 4 *p* = 0.024 (*)
	≥18 years 4.6% (73)			Promotion of comfort 26.0% (19)	
Geographic area of origin vs. Diagnoses	Sub-Saharan Africa 7.3% (86)	2.4 (1.6–3.6)	χ² = 19.99 df = 1 *p* < 0.0001 (*)	Headaches 25.8% (8)	χ² = 7.68 df = 4 *p* = 0.103 (ns)
	Maghreb 3.0% (31)			Gynecological 24.4% (21)	
Geographic area of origin vs. Nursing Interventions	Sub-Saharan Africa 6.5% (76)	2.3 (1.5–3.6)	χ² = 16.91 df = 1 *p* < 0.0001 (*)	Skin/wound care 32.1% (9)	χ² = 6.21 df = 4 *p* = 0.183 (ns)
	Maghreb 2.7% (28)			Promotion of comfort 23.7% (18)	
Seasonal time of the year vs. Diagnoses	Spring 6.0% (25)	n.a.	χ² = 11.82 df = 3 *p* = 0.0262 (*)	Dermatological 24.0% (6)	χ² = 13.6 df = 12 *p* = 0.323 (ns)
	Summer 6.8% (52)			Gynecological 19.2% (10)	
	Autumn 2.9% (20)			Dermatological 40.0% (8)	
	Winter 5.5% (20)			Dermatological 30.0% (6)	
Seasonal time of the year vs. Nursing Interventions	Spring 5.3% (22)	n.a.	χ² = 9.24 df = 3 *p* = 0.0080 (*)	Promotion of comfort 31.8% (7)	χ² = 21.36 df = 12 *p* = 0.0452 (*)
	Summer 6.0% (46)			Promotion of comfort 23.9% (11)	
	Autumn 2.9% (20)			Skin/wound care 40.0% (8)	
	Winter 4.4% (16)			Skin/wound care 37.5% (6)	

df—degree of freedom; (*)—statistically significant; (ns)—not significant; n.a.—not applicable.

## References

[1] Fernández P.P., Rubio J.F.I. (2008). Población extranjera y política de inmigración en Andalucía. Polít. Soc. (Madr.).

[2] Godenau D., Zapata-Hernández V.M. (2008). Canarias: Inmigración en una región fronteriza del sur de la Unión Europea. Polít. Soc. (Madr.)..

[3] Marcu S. (2010). Del Este Al Oeste: Geopolítica Fronteriza E Inmigración De La Europa Oriental A España.

[4] Fernández-Jurado J.A., Sabariego-Rivero S. (2006). Servicio Integral De Vigilancia Exterior (S.I.V.E). Consecuencias De Su Implantación. Boletín Criminológico Instituto Andaluz Interuniversitario De Criminología. https://bit.ly/2XVdfoY.

[5] European Union Agency for Fundamentals Rights (2011). Los Derechos Fundamentales De Los Inmigrantes En Situación Irregular En La Unión Europea. https://bit.ly/2UC5qT8.

[6] Pedrero-García E., Leiva-Olivencia J.J., García-Castaño F.J., Kressova N. (2011). El Derecho A La Salud De Los Inmigrantes: Favoreciendo La Calidad De Vida A Través De Los Planes Integrales Para La Inmigración En Andalucía. Actas Del I Congreso Internacional Sobre Migraciones En Andalucía.

[7] Junta De Andalucía (2016). Consejería De Justicia E Interior. III Plan Integral Para La Inmigración En Andalucía. https://bit.ly/2Hx0wD4.

[8] Real Decreto-Ley 7/2018, De 27 De Julio, Sobre El Acceso Universal Al Sistema Nacional De Salud. Boletín Oficial Del Estado, no 183, (30 July 2018). https://bit.ly/2NT4aqK.

[9] (2018). Intervención Social-Cruz Roja. https://bit.ly/2CmVJAC.

[10] Islam F., Khanlou N., Tamim H. (2014). South Asian populations in Canada: Migration and mental health. BMC Psychiatry.

[11] Toselli S., Gualdi-Russo E., Marzouk D., Sundquist J., Sundquist K. (2014). Psychosocial health among immigrants in central and southern Europe. Eur. J. Public Health.

[12] Jurado D., Alarcón R.D., Martínez-Ortega J.M., Mendieta-Marichal Y., Gutiérrez-Rojas L., Gurpegui M. (2017). Factores asociados a malestar psicológico o trastornos mentales comunes en poblaciones migrantes a lo largo del mundo. Rev. Psiquiatr. Salud Ment..

[13] Agudelo-Suárez A.A., Ronda-Pérez E., Gil-González D., Vives-Cases C., García A.M., García-Benavides F., Ruiz-Frutos C., López-Jacob M.J.M., Porthé V., Sousa E. (2009). Proceso migratorio, condiciones laborales y salud en trabajadores inmigrantes en España (proyecto ITSAL). Gac. Sanit..

[14] Hernández-Sánchez M.J., Segura-Clavell J., Burillo-Putze G. (2008). Papel clave de los servicios de emergencias en la tragedia de la inmigración ilegal por vía marítima. Emergencias.

[15] Matos-Castro S., Padrón-Peña M.P. (2008). Necesidades de asistencia urgente a los inmigrantes ilegales recién llegados en cayuco a Tenerife. Emergencias.

[16] Rodríguez del Rosario C., Núñez-Díaz S., García de Carlos P., Rodríguez-Palmero I., VMahtani-Mahtani V., Hernández-Rodríguez M.A. (2008). Características de la asistencia sanitaria a la llegada de inmigrantes africanos a las islas canarias. Emergencias.

[17] Norambuena-Coloma K., Mendoza-Parra S. (2008). Los desafíos en salud del inmigrante para la enfermería profesional. Enfermería Global.

[18] Ministerio De Sanidad, Servicios Sociales E Igualdad (2018). Clasificación Internacional De Enfermedades 10.ª Revisión. https://bit.ly/2W3ERXh.

[19] Butcher H., Bulechek G., Dochterman J.M., Wagne C. (2018). Clasificación De Intervenciones De Enfermería (NIC).

[20] World Medical Association (2013). Declaración De Helsinki De La AMM. https://bit.ly/2q7WZDo.

[21] Argimon-Pallàs J.M., Jiménez-Villa J. (2012). Métodos De Investigación Clínica Y Epidemiológica.

[22] Bará-Viñas J., López-Calero P., Villanueva-López C., Cueto-Acero M., Tabonet J., Dorrego-González T., Jiménez Antón P., Diez Medrano C. (2011). Migraciones Africanas Hacia Europa. Estudio Cuantitativo Y Comparativo. Años 2006–2008. https://bit.ly/1TWE7PP.

[23] Castilla-Vázquez C. (2017). Mujeres en transición: La inmigración femenina Africana en españa. Migr. Int..

[24] López-Domene E., Granero-Molina J., Fernández-Sola C., Hernández-Padilla J.M., López-Rodríguez M.D.M., Fernández-Medina I.M., Guerra-Martín M.D., Jiménez-Lasserrrotte M.D. (2019). Emergency care for women irregular migrants who arrive in spain by small boat: A qualitative study. Int. J. Environ. Res. Public Health.

[25] Jiménez-Lasserrotte M.M., López-Domene E., Fernández-Sola C., Hernández-Padilla J.M., Fernández-Medina I.M., Granero-Molina J. (2020). Accompanied child irregular migrants who arrive to Spain in small boats: Experiences and health needs. Glob. Public Health.

[26] Keim S.M., Mays M.Z., Parks B., Pytlak E., Harris R.M., Kent M.A. (2006). Estimating the incidence of heat-related deaths among immigrants in Pima County, Arizona. J. Immigr. Minority Health.

[27] Ruttan T., Stolz U., Jackson-Vance S., Parks B., Keim S.M. (2013). Validation of a temperature prediction model for heat deaths in undocumented border crossers. J. Immigr. Minority Health.

[28] Vélez-Alcalde F.J. (2008). Pateras, Cayucos Y Mafias Transfronterizas En África: El Negocio De Las Rutas Atlánticas Hacia Las Islas Canarias (ARI). https://bit.ly/2UBbM4Z.

[29] Cattaneo C., Tidball Binz M., Penados L., Prieto J., Finegan O., Grandi M. (2015). The forgotten tragedy of unidentified dead in the Mediterranean. Forensic Sci. Int..

[30] Boyce M.R., Katz R., Standley C.J. (2019). Risk factors for infectious diseases in urban environments of sub-saharan Africa: A systematic review and critical appraisal of evidence. Trop. Med. Infect. Dis..

[31] Loreto-Cid M. (2014). Cefaleas, evaluación y manejo inicial. Rev. Med. Clin. Condes.

[32] Zamatto F., Argenziano S., Arsenijevic J., Ponthieu A., Bertotto M., Di Donna F., Harries A.D., Zachariah R. (2017). Migrants caught between tides and politics in the Mediterranean: An imperative for search and rescue at sea?. BMJ Glob. Health.

[33] Di Meco E., Di Napoli A., Amato L.A., Fortino A., Costanzo G., Rossi A., Mirisola C., Petrelli A., INMP Team (2018). The INMP Team. Infectious and dermatological diseases among arriving migrants on the Italian coasts. Eur. J. Public Health.

[34] Dorn T., Ceelen M., Tang M.J., Browne J.L., de Keijzer K.J., Buster M.C., Das K. (2011). Health care seeking among detained undocumented migrants: A cross-sectional study. BMC Public Health.

[35] Chen W., Hall B.J., Ling L., Renzaho A.M.N. (2017). Pre-migration and post-migration factors associated with mental health in humanitarian migrants in Australia and themoderation effect of post-migration stressors: Findings from the first wave data of the BNLA cohort study. Lancet Psychiatry.

[36] Buron A., Cots F., Garcia O., Vall O., Castells X. (2008). Hospital emergency department utilisation rates among the immigrant population in Barcelona, Spain. BMC Health Serv. Res..

[37] Norredam M., Nielsen S.S., Krasnik A. (2010). Migrants’ utilization of somatic healthcare services in Europe. A systematic review. Eur. J. Public Health.

[38] Rué M., Cabré X., Soler-González J., Bosch A., Almirall M., Serna M.C. (2008). Emergency hospital services utilization in Lleida (Spain): A cross-sectional study of immigrant and Spanish-born populations. BMC Health Serv. Res..

[39] Helgesson M., Johansson B., Nordquist T., Vingård E., Svartengren M. (2019). Healthy migrant effect in the Swedish context: A register-based, longitudinal cohort study. BMJ Open.

[40] Leppälä S., Lamminpää R., Gissler M., Vehviläinen-Julkunen K. (2019). Hindrances and facilitators in humanitarian migrants’ maternity care in Finland: Qualitative study applying the three delays model framework. Scand. J. Caring Sci..

[41] Sheikh S. (2019). Risk factors associated with emergency department recidivism in the older adult. West. J. Emerg. Med..

[42] Elstad J.I. (2016). Register study of migrants’ hospitalization in Norway: World region origin, reason for migration, and length of stay. BMC Health Serv. Res..

[43] Alvarez-Leiva C. (2012). Asistencia sanitaria a múltiples víctimas y catástrofes. Sevilla: Samu Editorial.

